# Reply to: Response to the Union’s statement on ongoing conflicts and impact on health services

**DOI:** 10.5588/ijtldopen.26.0311

**Published:** 2026-10-12

**Authors:** Z. Mor, A. Rokach, O. Wand, M. Borow, Z.G. Fridlender, B.D. Fox

**Affiliations:** 1 Jerusalem Department of Health, Ministry of Health, Jerusalem, Israel;; 2 School of Health Sciences, Ashkelon Academic College, Ashkelon, Israel;; 3 Israeli Lung and Tuberculosis Association, Tel Aviv, Israel;; 4 Department of Internal Medicine, Pulmonary Institute, Shaarei Zedek Medical Center, Jerusalem, Israel;; 5 Hadassah–Hebrew University School of Medicine, Jerusalem, Israel;; 6 Pulmonary Department, Barzilay Medical Center, Ashkelon, Israel;; 7 Division of Law and Policy, Israeli Medical Association, Ramat Gan, Israel;; 8 Department of Internal Medicine, Hadassah Ein Karem, Jerusalem, Israel;; 9 The Israeli Society of Pulmonary Medicine, Ashkelon, Israel;; 10 Pulmonary Medicine Institute, Shamir Medical Center, Zrifin, Israel;; 11 Grey Faculty of Medicine, Tel Aviv University, Tel Aviv, Israel.

**Keywords:** tuberculosis, Israel, Gaza, conflict, public health

Dear Editor,

We share the authors’ concern for all innocent people, both in Israel and in Gaza.^1^ However, their Correspondence,^1^ which refers to The Union Statement on Ongoing Conflicts and the Impact on Health Services,^2^ makes unsubstantiated allegations about the ‘silence’ of medical professional organisations, while conspicuously ignoring crucial facts regarding the conflict. Most notably, the Correspondence does not address any reference to Hamas’s well-documented terrorist attacks, particularly its massacre on 7 October 2023, and their related atrocities.^3^

As a basis, it is important to note that Israel withdrew entirely from Gaza in 2005, giving Gazans the chance to build their future and construct a robust health care system. Tragically for both Gazans and Israelis, Hamas was elected democratically in 2007, on a platform calling for Israel’s eradication. Since 2007, Hamas has fired tens of thousands of rockets into Israel, targeting innocent citizens and Israeli hospitals, while Israel took in sick Gazans to receive medical care and permitted health care workers to receive training in Israel.^4^

On 7 October 2023, Hamas and other terror organisations breached the Israeli–Gaza security fence, invaded Israeli territory, and took control of over 25 towns and villages. Terrorists from Hamas and other organisations broke into civilian homes and military bases and murdered more than 1,200 civilians and soldiers, some of whom were tortured or raped before or after death.^5^ Of 816 civilians murdered, ranging in age from 5 months to 91 years, 87.4% were Jews, 1.6% Arabs, 8.5% work migrants, and 2.6% whose citizenship or religion could not be determined. Hamas took more than 250 hostages to Gaza, mostly civilians (Jews, Christians, Muslims, and Buddhists). The hostages were tortured, starved, and held underground for up to 2 years under inhuman conditions, with no concern for their medical conditions, including significant injuries inflicted during the October 7 attack and pre-existing lung or other diseases.^6–8^ By contrast, Gazan terrorist detainees received medical treatment in a dedicated field hospital staffed by highly professional, well-regarded physicians and novel technologies not yet available in many civilian hospitals.^9^ Meanwhile, hospitals in Gaza were documented as sites for weapon storage, rocket launches, and hostage detention (causing them to lose their protected status under the Geneva Convention).^10^ One hostage was even murdered *inside* a Gazan hospital.^11^

With reference to TB, the Gaza Strip is a low-TB-burden region with an annual incidence of 3.5 cases per 100,000 population. In 2022, two TB cases were registered in Gaza^12^ and none in 2023.^13^ Israel is also a low-TB-burden country, with similar incidence in the native Arab and Jewish population and higher incidence amongst immigrants.^14^ Between March and December 2025, Israel facilitated the entry of more than 200 packs of isoniazid, despite a general closure. These facts are not reflected in the Correspondence. In August 2024, when a verified case of vaccine-derived poliovirus type 2 (cVDPV2) was diagnosed in a 10-month-old girl, Israel allowed entry of novel oral polio vaccine type 2 (nOPV2) to enable an international vaccination campaign reaching more than 600,000 children.^15^ The two-round campaign was conducted during negotiated pauses in fighting.^16^

The facts we have provided stand completely at odds with the allegations made by the authors of the Correspondence. We echo the authors’ call for solidarity with health care workers under threat, but solidarity must be grounded in accuracy, not selective narrative. The welfare of Gazan civilians is a legitimate and urgent concern, but so too is the acknowledgment that the governing authority of Gaza bears responsibility for those civilians’ conditions. Hamas’s founding aspiration, the elimination of Israel and the killing of people from Israel and other nationalities, remains an obstacle not only to peace but to the very humanitarian outcomes the authors claim to want.
